# The Effects of Repeat Testing, Malingering, and Traumatic Brain Injury on Computerized Measures of Visuospatial Memory Span

**DOI:** 10.3389/fnhum.2015.00690

**Published:** 2016-01-05

**Authors:** David L. Woods, John M. Wyma, Timothy J. Herron, E. W. Yund

**Affiliations:** ^1^Human Cognitive Neurophysiology Laboratory, Veterans Affairs Northern California Health Care System, Martinez, CAUSA; ^2^Department of Neurology, University of California, Davis, Sacramento, CAUSA; ^3^Center for Neuroscience, University of California, Davis, Davis, CAUSA; ^4^Center for Mind and Brain, University of California, Davis, Davis, CAUSA

**Keywords:** digit span, reaction time, concussion, feigning, head injury, reliability, computer

## Abstract

Spatial span tests (SSTs) such as the Corsi Block Test (CBT) and the SST of the Wechsler Memory Scale are widely used to assess deficits in spatial working memory. We conducted three experiments to evaluate the test–retest reliability and clinical sensitivity of a new computerized spatial span test (C-SST) that incorporates psychophysical methods to improve the precision of spatial span measurement. In Experiment 1, we analyzed C-SST test–retest reliability in 49 participants who underwent three test sessions at weekly intervals. Intraclass correlation coefficients (ICC) were higher for a psychophysically derived mean span (MnS) metric (0.83) than for the maximal span and total correct metrics used in traditional spatial-span tests. Response times (ReTs) also showed high ICCs (0.93) that correlated negatively with MnS scores and correlated positively with response-time latencies from other tests of processing speed. Learning effects were insignificant. Experiment 2 examined the performance of Experiment 1 participants when instructed to feign symptoms of traumatic brain injury (TBI): 57% showed abnormal MnS z-scores. A MnS z-score cutoff of 3.0 correctly classified 36% of simulated malingerers and 91% of the subgroup of 11 control participants with abnormal spans. Malingerers also made more substitution errors than control participants with abnormal spans (sensitivity = 43%, specificity = 91%). In addition, malingerers showed no evidence of ReT slowing, in contrast to significant abnormalities seen on other malingered tests of processing speed. As a result, differences between ReT z-scores and z-scores on other processing speed tests showed very high sensitivity and specificity in distinguishing malingering and control participants with either normal or abnormal spans. Experiment 3 examined C-SST performance in a group of patients with predominantly mild TBI: neither MnS nor ReT z-scores showed significant group-level abnormalities. The C-SST improves the reliability and sensitivity of spatial span testing, can accurately detect malingering, and shows that visuospatial working memory is largely preserved in patients with predominantly mild TBI.

## Introduction

Spatial span tests (SSTs) have been widely used to evaluate visuospatial memory in patients with such diverse disorders as dementia (Wiechmann et al., 2011), schizophrenia (Chey et al., 2002), Parkinson’s disease (Yaguez et al., 2006), stroke (Kessels et al., 2000), PTSD (Flaks et al., 2014), and ADHD (Yang et al., 2011). The most familiar SST is the Corsi Block Test (CBT) (Berch et al., 1998), in which the participant is presented with a set of nine blocks fixed on a board. The blocks are tapped in sequence by the examiner, beginning with a sequence length of two blocks, and the participant repeats the tapping sequence in the same order. Two trials are tested at each length, with trial length increasing if the participant reproduces either or both of the two sequences correctly. Testing ceases when the participant misses both trials of the same length. Maximal span is quantified as the length of the longest sequence correctly reproduced. A similar test procedure is used in the Wechsler Memory Scale (WMS) with a 10-block layout (Wechsler, 1997). However, instead of quantifying maximal span, the sum of the number of correct trials is recorded (Wechsler, 1997; Wilde et al., 2004; Lo et al., 2012).

In a companion manuscript (Woods et al., 2015b), we described the rationale for the development of a new computerized version of the SST (C-SST) that differs in three major respects from traditional SSTs. First, the C-SST randomizes both the spatial display and trial sequence on each trial. Second, the C-SST presents sequences at list lengths below and above maximal span, using a staircase procedure to estimate mean span (MnS), a psychophysical metric that shows lower measurement variance in comparison with the maximal span and total correct metrics used in conventional SSTs. Finally, the C-SST accurately measures response times (ReTs), the average time needed to select each square in the sequence.

In the companion study, we evaluated the effects of demographic variables, including age, sex, education, and computer-use, on C-SST performance in a normative control sample of 187 participants ranging in age from 18 to 82 years. Experimental results showed that age and computer-use accounted for much of the performance variance, and simulations of the results obtained with different tests (e.g., CBT, Wechsler SST, and C-SST) showed that the MnS metric provided the most accurate estimate of “true span” (the span length where a subject would correctly report 50% of trials).

Here, we describe three experiments using the C-SST. In Experiment 1, 49 young control participants underwent three test sessions at weekly intervals. Experiment 1a was used to evaluate the goodness-of-fit of the regression functions defined in the normative study. Experiments 1b and 1c permitted the analysis of learning effects and test–retest reliability. In Experiment 2, the participants from Experiment 1 were instructed to feign symptoms of traumatic brain injury (TBI). The goal of Experiment 2 was to quantify the effects of malingering on performance and to evaluate malingering indices that could assist in determining whether abnormal spatial span scores were due to malingering. Finally, in Experiment 3, we examined performance in patients with TBI of varying severity. In all three experiments, we used multiple linear regression based on the regression functions from the previous normative population to estimate MnS and ReT z-scores that were corrected for the contributions of age and computer-use.

## Experiment 1: Test–Retest Reliability

In Experiment 1, we studied a group of 49 young participants who underwent three test sessions at weekly intervals to examine test–retest reliability and learning effects. Although the test–retest reliability of the CBT maximal span has not previously been measured directly, Park et al. (2002) found high intraclass correlation coefficients (ICC, 0.79) in split-half comparisons of forward and backward CBT spans measured during the same test session. Lo et al. (2012) found moderate test–retest reliability (*r* = 0.46 to *r* = 0.66) in the total correct metric of the Wechsler Spatial Span Test in different age cohorts tested at intervals of 2–7 years, and also reported insignificant learning effects. Based on the absence of learning effects in tests where both the block layout and the path sequences are repeated over tests, we anticipated minimal learning on the C-SST, where both the display layout and path sequences changed randomly on each trial.

### Methods

#### Participants

Forty-nine young participants (mean 26.3 years, range 18–46 years, 53% male) were recruited from existing subject pools and from online advertisements on Craigslist. Young participants were selected from those who volunteered to undergo four test sessions. The C-SST was administered midway through the California Cognitive Assessment Battery (CCAB)^1^ and required approximately 5 min to complete. The results of other CCAB tests from the same participant population have previously been reported for digit span (Woods et al., 2011a), finger tapping (Hubel et al., 2013), simple reaction time (Woods et al., 2015c), choice reaction time (Woods et al., 2015e), question completion time (Woods et al., 2015g), and trail making (Woods et al., 2015d).

Participants were required to meet the following inclusion criteria: (a) fluency in English; (b) no history of psychiatric or neurological disease; (c) no current substance abuse; (d) no history of hospitalization for head trauma; (e) on a stable dosage of any required medication; (f) auditory functioning sufficient to understanding normal conversational speech; and (g) visual acuity normal or corrected to 20*/*40 or better. Participants volunteered to take part in three weekly test sessions to evaluate test-retest reliability, and in a fourth session to study the effects of malingering (see Experiment 2, below). Experiment 1 participants were primarily college students who were younger [*p* < 0.01] and reported higher levels of computer-use [*p* < 0.03] than the participants in the normative population, as shown in **Table 1**. Ethnically, 68% of the participants were Caucasian, 11% Latino, 9% African American, 10% Asian, and 2% other. All participants gave informed written consent following procedures approved by the Institutional Review Board of the VA Northern California Health Care System (VANCHCS) and were paid for their participation.

**Table 1:** Participants in the experiments. Range, mean, and variance are shown for age and education.

Experiment	Group	*N*	Age (years)	Education (years)	C-use scale	Male (%)
Normative	Control	187	18–82; 41.1 (21.3)	10–20; 14.6(2.0)	5.12	58%
Experiment 1/2	Control/Malinger	49	18–46; 26.3 (5.9)	12–18; 14.6 (1.5)	5.92	53%
Experiment 3	TBI	28	20–61; 33.4 (11.0)	12–18; 13.6 (1.45)	5.16	96%


C-use, computer-use. Normative data from Woods et al. (2015b).

#### Apparatus and Stimuli

The methods were identical to those used during normative data collection (Woods et al., 2015b). Testing was performed in a quiet room using a standard Windows desktop computer running Presentation software (Versions 13 and 14, NeuroBehavioral Systems, Albany, CA, USA)^2^. Ten red squares were presented on each trial, with five squares presented in each hemifield. The display was divided into 10 rows and 10 columns (five squares in each hemifield). A single square was placed in each horizontal row and vertical column, with potential locations randomly filled in each hemifield. Small and random horizontal and vertical offsets of each square prevented squares from being aligned. Thus, in contrast to typical spatial span tasks, where the same block layout is used across all trials and participants, different displays were presented on each trial for each participant in the C-SST. In addition, sequences were selected randomly, with the constraint that a square could only be included once in each sequence.

**FIGURE 1:**
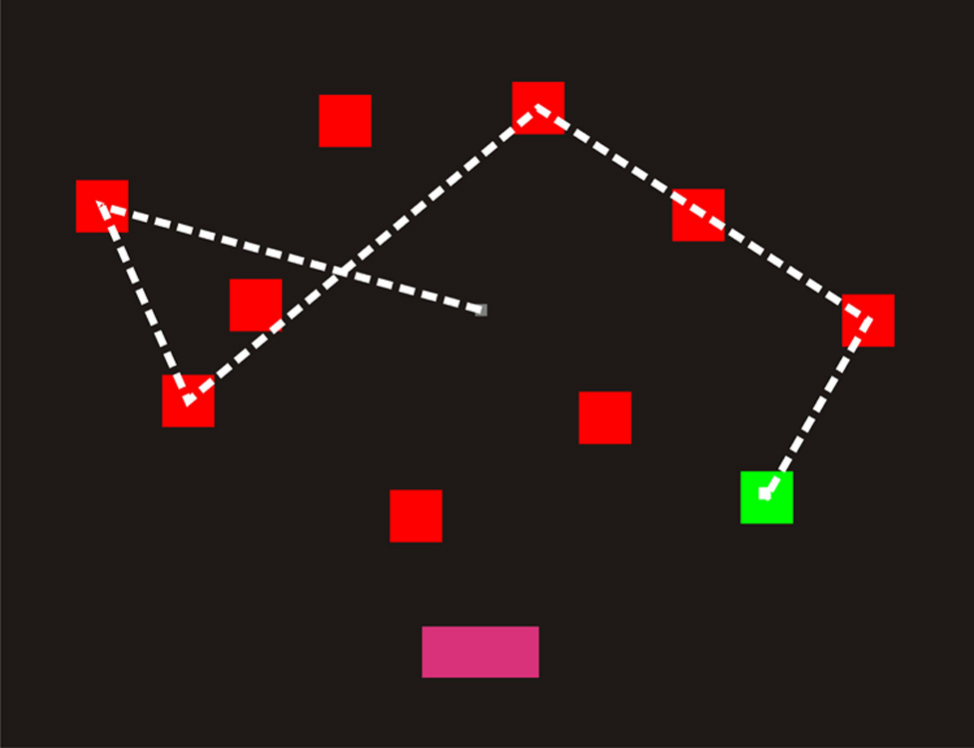
**A computerized spatial span test (C-SST) sequence of length five.** The square sequence was shown to participants using the cursor (a small white square), which moved from square to square over 977 ms intervals. Each square flashed green as it was selected (bottom right). When the sequence had been shown, the “Done” button illuminated (bottom center). The participant’s task was to select the squares in the correct order using the mouse. The dashed line has been included to illustrate the cursor displacements, but was not visible to participants. Participants clicked “Done” when their selection was complete.

Four practice trials were followed by 14 test trials. The display for the practice trials contained six squares (three in each hemifield). Practice testing began with two-square sequences and included feedback. Test trials did not include feedback and began at a sequence length of three. Sequence length increased by one following each correct trial and decreased by one following two successive misses. As shown in **Figure 1**, the program selected squares by moving the white cursor to each chosen square over a period of 677 ms, followed by a 300 ms period when the selected square flashed green–red–green (100 ms each), before finally returning to red to indicate that a selection had been made. When sequence selection was complete, the cursor returned to the center of the display and a “DONE” button appeared at the bottom of the screen. During the response phase, the participant used a computer gaming mouse (Razer Sidewinder, Carlsbad, CA, USA) to control the cursor and selected each square by pressing the left mouse button. ReTs, the average time to select each square, were recorded (in ms) for each participant. When finished, the participant clicked the “Done” button, whereupon a blank display with a “Next” button appeared.

##### Scoring Metrics

The data from individual trials were analyzed using different automated scoring metrics similar to those used in the companion study (Woods et al., 2015b), including maximal span from the Corsi Block Test, maximal length (the longest correct trial among all 14 trials), the mean sequence length of the last five trials, and the total number of correct trials (similar to the Wechsler SST). In addition, we estimated MnS, the extrapolated list length where 50% of sequences would be correctly reported, based on psychophysical estimation (Killion et al., 2004). The MnS baseline was set at 2.5 blocks and was incremented by the fraction of the trials accurately reported at each succeeding list length. Finally, we measured mean ReTs averaged over the item selections for all 14 trials. Only two variables in the previous normative study (Woods et al., 2015b), age and computer-use, had significant influences on MnS and log-transformed ReT values. Therefore, *z*-scores for MnS and log-ReT were created using the multiple regression functions derived from the normative data set, specifically: MnS = 5.68 −0.022∗ Age + 0.093∗ Computer-use; ReT = 1560 + 8.16∗ Age – 62.3∗ Computer-use.

#### Statistical Analysis

We used analysis of variance (ANOVA) for repeated measures to analyze changes in the different measures. Greenhouse–Geisser corrections of degrees of freedom were uniformly used in computing *p*-values in order to correct for covariation within factors or interactions. Effect sizes are reported as partial ω^2^ values. ICCs and power analyses are included for significant results. Ninety five percent confidence ranges for correlation coefficients were calculated using SPSS (version 22).

## Results

**FIGURE 2:**
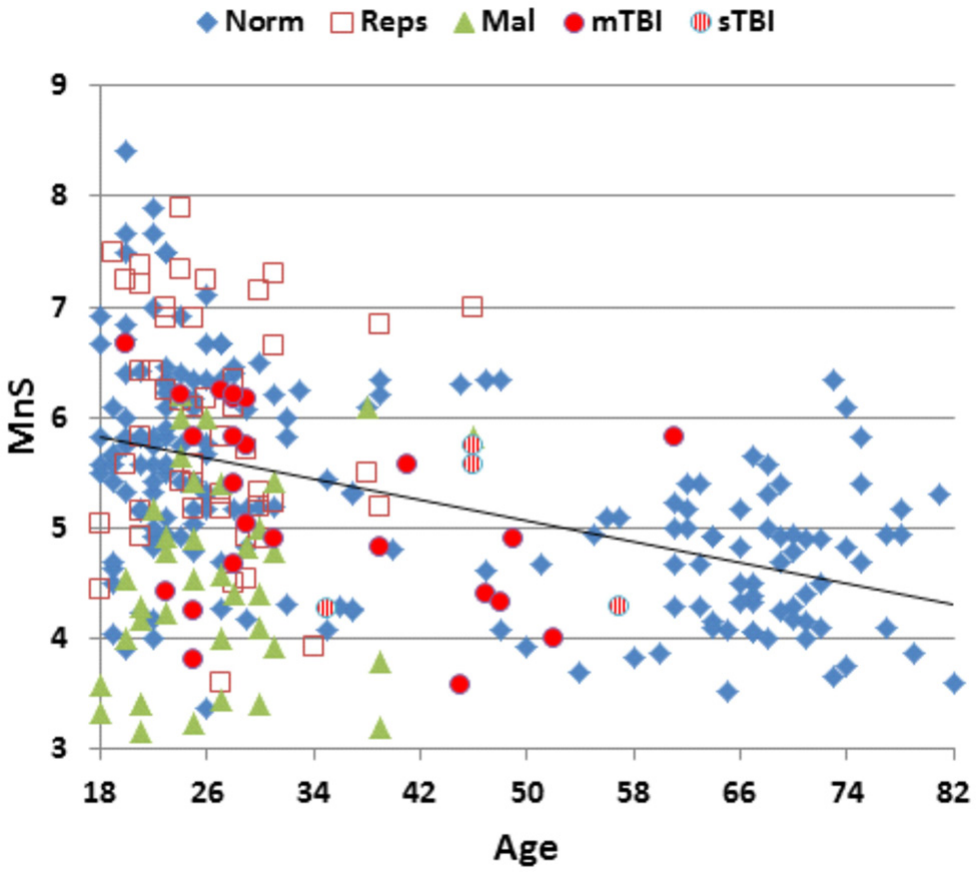
**Mean spatial span (MnS) of participants as a function of age.** For control participants in the normative dataset (Norm.), participants in Experiment 1a (Reps), malingering participants (Mal, Experiment 2), and mild and severe TBI (mTBI and sTBI) patients (Experiment 3).

**FIGURE 3:**
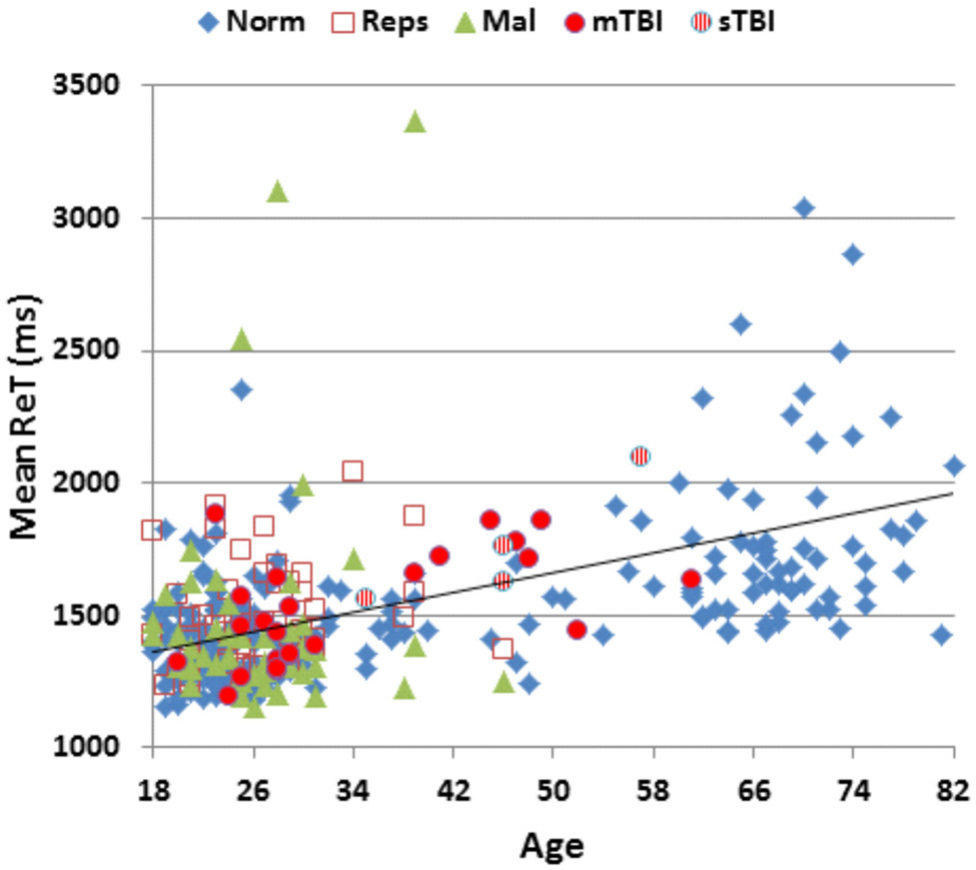
**Mean response times (ReTs) as a function of age.** ReTs were averaged over different list positions. See **Figure 2** for group membership specifications.

**Table 2** presents mean scores for the different metrics in the normative population and the three test sessions of Experiment 1. MnS scores for individual participants (open, red squares) from Experiment 1a are shown in **Figure 2** as a function of age, and mean ReTs for individual participants (open, red squares) are shown as a function of age in **Figure 3**. As in our previous study (Woods et al., 2015b), MnS scores had lower variance and a lower coefficient of variation than maximal span scores (see **Table 2**).

**Table 2:** Performance metrics in the different experiments.

	TotC	ML	MS	MnS	MnS-z	ReT (ms)	L-ReT-z
Normative	5.95 (0.92)	5.93 (1.01)	5.15 (1.20)	5.27 (1.01)	0.00 (1.00)	1742 (426)	0.00 (1.00)
E1a	6.58 (1.01)	6.51 (1.04)	5.73 (1.32)	5.95 (1.03)	0.34 (1.20)	1569 (199)	−0.01 (0.80)
E1b	6.52 (0.85)	6.57 (1.00)	5.88 (1.44)	6.04 (1.04)	0.45 (1.18)	1510 (188)	−0.25 (0.79)
E1c	6.60 (0.84)	6.71 (1.04)	5.80 (1.22)	6.09 (0.98)	0.50 (1.10)	1511 (251)	−0.28 (1.05)
E2. Mal	5.16 (1.11)	4.59 (1.19)	3.80 (1.19)	4.10 (1.18)	−1.82 (1.37)	1588 (493)	−0.09 (1.49)
E3. mTBI	5.88 (0.76)	6.00 (0.95)	5.04 (1.60)	5.21 (0.89)	−0.26 (0.99)	1702 (386)	−0.01 (0.80)
E3. sTBI	5.75 (0.50)	5.75 (0.96)	5.25 (0.96)	4.97 (0.80)	−0.16 (0.99)	2047 (180)	0.89 (0.31)


*TotC, total correct; ML, maximum length reported; MS, Corsi block span, maximum sequence length reported before missing two successive trials; MnS, mean spatial span; MnS-Z, age- and computer-use regressed MnS z-score; ReT, mean response time; L-ReT-z, z-score of age- and computer-use regressed, log transformed ReTs. Standard deviations are shown in parentheses.*

**FIGURE 4:**
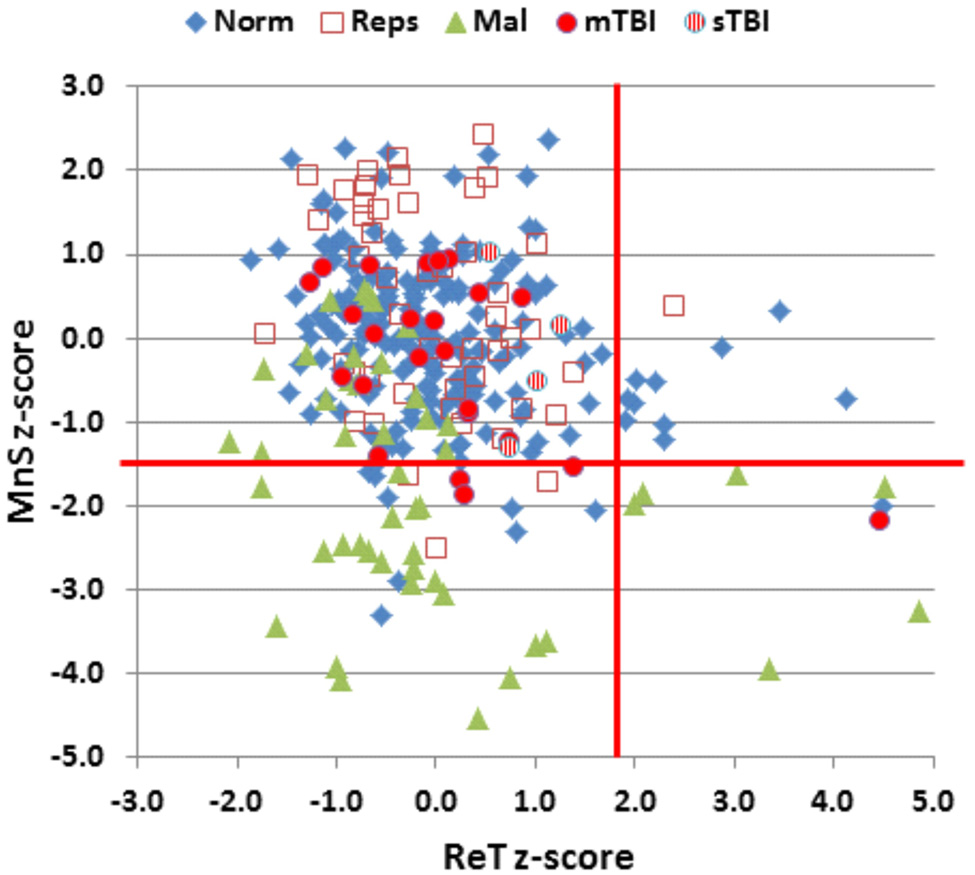
**Mean span (MnS) and log-transformed ReT *z*-scores.** The *z*-scores were corrected for the contributions of age and computer-use using multiple regression values from the normative data. Vertical and horizontal red lines show *p* < 0.05 abnormality thresholds. See **Figure 2** for group membership specifications.

The participants in Experiment 1 had greater MnS scores and faster response times than the participants in the previously tested normative population, due primarily to their younger age. **Figure 4** shows the MnS and ReT z-scores for individual participants after corrections for age and computer-use. MnS z-scores (mean = 0.34) in the first test session were slightly higher than those predicted from the age and computer-use regression functions obtained in the normative population, although the difference showed a very small effect size [*F*(1,234) = 4.17, *p* < 0.05, partial ω^2^ = 0.01]. Power analysis showed that 930 subjects would be required to have a 95% chance of obtaining a difference with the normative population at the *p* < 0.05 level. ReT *z*-scores (mean = −0.01) showed no significant differences from the normative data.

### Test–Retest Reliability

Intraclass correlation coefficients were high for MnS scores (0.84), log-ReT scores (0.94), MnS *z*-scores (0.83), and ReT *z*-scores (0.93). ICCs were lower for the maximal span (0.68) and total correct (0.67) metrics typically obtained in traditional SSTs.

### Learning Effects

Although MnS scores improved slightly (by 2.4%) over the repeated tests, omnibus learning effects failed to reach significance [*F*(2,96) = 0.64, NS]. No significant improvements were seen in response times [*F*(2,96) = 3.16, *p* < 0.06] or in any of the other metrics, as shown in **Table 2**.

## Discussion

As in the previous study of visuospatial span (Woods et al., 2015b), the MnS metric showed lower variance and a reduced coefficient of variation in comparison with the maximum span metric traditionally used in CBT studies. Similar results were also obtained in previous comparisons of traditional and MnS-like psychophysical measures of digit span (Woods et al., 2011a). This reflects the fact that the MnS metric estimates spatial span using contributions from all 14 trials and repeatedly samples spans above and below the maximal span. In contrast, the traditional maximal span and the Wechsler total correct metrics reflect only the trials presented before two trials are missed in a row. As a result, the traditional sequence-delivery rule increases variance and systematically underestimates the true spatial span (Woods et al., 2015b).

The participants in Experiment 1 had slightly better MnS *z*-scores than those of the normative population. However, the span difference (0.29 items) was considerably smaller than the differences in maximal spatial spans reported in previous large scale studies using the CBT (Berch et al., 1998). For example, in large normative studies (350+ participants each) with similar mean population ages (range 53.4 versus 57.2), Orsini et al. (1986) reported a maximal span of 4.56, while Monaco et al. (2013) reported a maximal span of 5.38.

Intraclass correlation coefficient estimates of reliability were higher for the MnS metric and MnS *z*-scores than for the traditional maximal span and the total correct metrics (Lo et al., 2012). ICCs were also high for the ReT *z*-score measure, demonstrating that both visuospatial span and response time were reliably measured in the C-SST paradigm.

We found no significant learning effects in repeated spatial span testing. This result was not surprising, as insignificant learning effects have previously been reported on the WMS SST, which uses a fixed block display and identical sequences for all tests (Lo et al., 2012). Learning in the C-SST would presumably be more difficult because the block display changes on each trial and the list sequences are selected randomly on repeated tests.

## Experiment 2: Simulated Malingering

When a patient’s SST results fall into the abnormal range, the examiner is faced with the challenge of determining whether impaired performance is due to organic causes or suboptimal effort. The detection of malingering is particularly important in the evaluation of patients with head injury, where litigation and pension claims often provide financial incentives to perform with suboptimal effort (Armistead-Jehle, 2010). Therefore, performance validity tests such as the Test of Memory Malingering (Tombaugh, 1996) are widely used to evaluate whether a participant is performing with full effort. In addition to the standalone performance validity tests, performance validity metrics (PVMs), embedded measures such as reliable digit span (Babikian et al., 2006), can be extracted from established cognitive tests, and multiple PVMs can provide a reliable measure of suboptimal effort (Larrabee, 2014).

Previous studies have demonstrated decrements in spatial span in control participants who were instructed to malinger (Bernard, 1990), and in patients who are thought to be malingering based on the existence of pension or disability claims and evidence of malingering on performance-validity tests (Ord et al., 2008). This has led to efforts to develop PVMs based on SST performance. For example, Ylioja et al. (2009) developed a “reliable spatial span” metric for the Wechsler SST by summing the longest forward and backward spans repeated correctly on two successive trials of the same length. They found that a reliable spatial span of six accurately classified 55% of malingerers and 86% of non-malingerers. Studies using analogous reliable digit span measures have reported similar classification accuracies, showing a sensitivity of 60% in detecting malingerers with a specificity of 89% (Greve et al., 2007). However, in the same study, normal digit-span score cutoffs alone accurately classified 58% of malingering subjects with a specificity of 89%, i.e., the majority of malingering subjects also had significantly abnormal digit span scores.

In clinical practice, patients with test scores that fall within the normal range are rarely evaluated for signs of malingering. Rather, examiners are more focused on determining whether abnormal test scores are due to malingering or organic impairment. Therefore, in Experiment 2, we evaluated malingering detection using both standard comparisons of malingering participants and controls and additional comparisons of malingering participants with abnormal scores and control participants with abnormal scores. We investigated how well three PVMs could identify malingering participants: (1) *z*-score cutoffs: We hypothesized that malingering participants would have a greater incidence of abnormal MnS scores, and that malingering participants with abnormal scores would produce abnormalities of greater magnitude than controls with abnormal scores. (2) Types of errors: On digit span tests, malingering participants produce different patterns of errors than control participants (Woods et al., 2011b). Therefore, we investigated whether the types of errors (e.g., transposition, substitution, addition, permutation, omission) could identify malingering participants in the C-SST. (3) Response times: ReT *z*-scores in control participants were negatively correlated with MnS *z*-scores (*r* = −0.27), i.e., participants with poorer spans took longer to respond. In addition, ReT *z*-scores were positively correlated with *z*-score measures of processing speed on other tests performed on the same day, including simple reaction time (*r* = 0.17) (Woods et al., 2015a), choice reaction time (*r* = 0.21) (Woods et al., 2015e), the Trail Making Test, Part A (*r* = 0.39) (Woods et al., 2015d), and question completion time (*r* = 0.44) (Woods et al., 2015g). Since malingering subjects have difficulty equating the magnitude of deficits on different processing speed tests (Kertzman et al., 2006; Woods et al., 2015f), we compared ReT *z*-scores with the results of other tests of processing speed obtained during simulated malingering.

### Methods

#### Participants and Methods

With the exception of the instructions given to participants, the methods were identical to those used in the first session of Experiment 1. The 49 participants of Experiment 1 were given written instructions after the third test session to perform like a patient with a minor head injury when participating in a fourth test session during the following week. The additional instructions were as follows: “Listed below you’ll find some of the symptoms common after minor head injuries. Please study the list below and develop a plan to fake some of the impairments typical of head injury when you take the next test. Do your best to make your deficit look realistic. If you make too many obvious mistakes, we’ll know you’re faking! Symptom list: Difficulty concentrating for long periods of time, easily distracted by unimportant things, headaches and fatigue (feeling “mentally exhausted”), trouble coming up with the right word, poor memory, difficulty performing complicated tasks, easily tired, repeating things several times without realizing it, slow reaction times, trouble focusing on two things at once.”

### Results

Mean performance measures for malingering participants are included in **Table 2**. **Figure 2** shows MnS scores (green triangles) and **Figure 3** shows ReTs from malingering participants (green triangles) as a function of age. All span metrics were reduced in malingering conditions. However, the sensitivity of different metrics varied considerably. Greater sensitivity to malingering was seen for MnS measures (28 abnormal scores) in comparison to maximal span or total correct measures (seven abnormal scores each). Statistical comparisons showed that malingering participants as a group produced lower MnS z-scores than those of the normative control group [MnS *z*-score = −1.81, *F*(1,234) = 108,3, *p* < 0.0001, partial ω^2^ = 0.31]. There were also significant *z*-score decrements in comparison with full-effort conditions in Experiment 1a [*F*(1,48) = 78.29, *p* < 0.0001, partial ω^2^ = 0.62]. Power analysis showed that ten subjects would be required to have a 95% chance of obtaining a difference at the *p* < 0.05 level.

**Figure 4** shows the MnS and ReT z-scores from malingering participants (green triangles). Among the participants instructed to malinger, 57% produced MnS *z*-scores in the abnormal range (*p* < 0.05, *z*-score = −1.50). As a result, a *z*-score cutoff of −1.5 showed a sensitivity of 57% and a specificity of 95% in distinguishing malingering participants from controls. When only those control participants with abnormal *z*-scores (i.e., nine normative controls and three subjects from Experiment 1a) were compared with malingering participants with abnormal MnS *z*-scores, greater *z*-score abnormalities were seen in the malingering group. For example, a *z*-score cutoff of 3.0 accurately categorized 36% of malingering participants and 91% of controls with abnormal spans, while a *z*-score cutoff of 2.5 accurately classified 55% of malingering participants and 73% of controls with abnormal spans.

**FIGURE 5:**
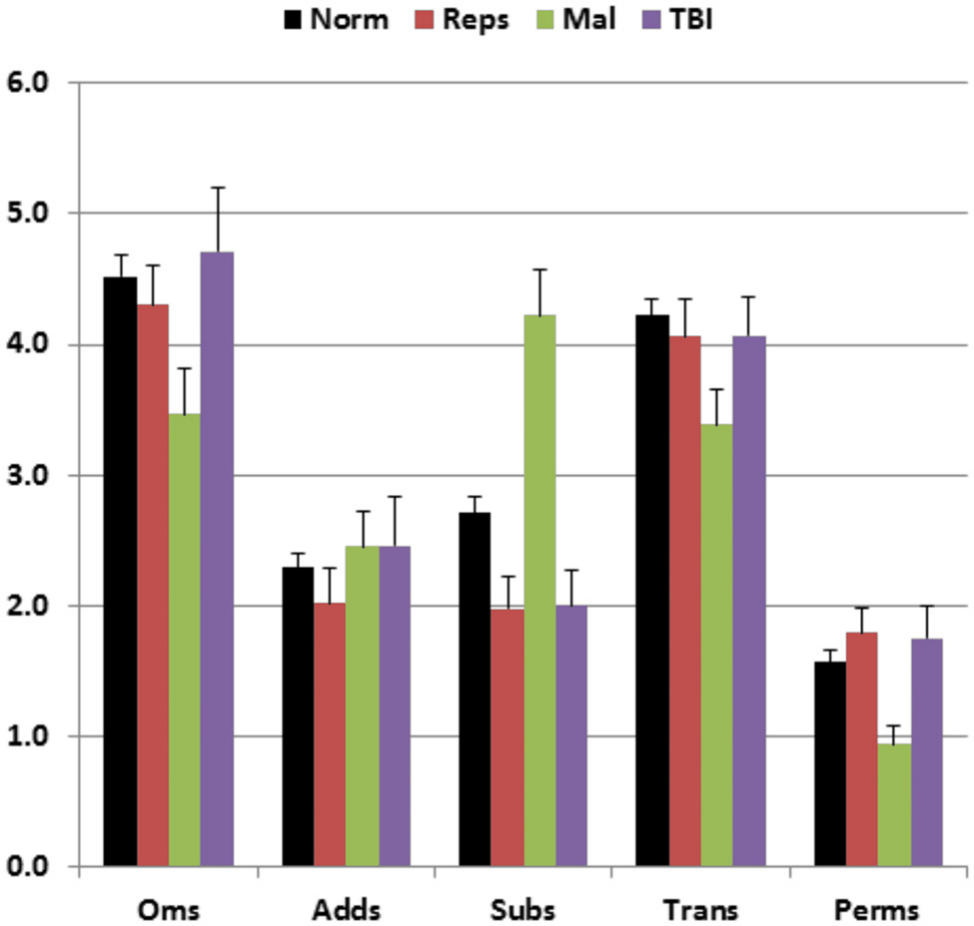
**Percentage of errors of different types made by participants of different groups.** Oms, omissions; Adds, additions; Subs, substitutions; Trans, transpositions; Perms, permutations. Error bars show standard errors. See **Figure 2** for group membership specifications.

**Figure 5** shows the types of errors made by different participant groups. Malingering participants (green bars) showed an increase in the relative incidence of substitution errors in comparison with both the normative controls (black bars) [*F*(1,234) = 33.07, *p* < 0.0001, partial ω^2^ = 0.12] and the participants in Experiment 1a (red bars) [*F*(1,48) = 29.90, *p* < 0.0001, partial ω^2^ = 0.38]. Overall, the percentage of substitution errors showed a sensitivity of 29% and a specificity of 95% in distinguishing malingering participants from controls. In addition, there was a strong negative correlation between MnS *z*-scores and the percentage of substitution errors among malingering participants [*r* = −0.47, *t*(47) = 3.46, *p* < 0.002] that was weaker in control subjects [*r* = −0.21 in the normative sample, and *r* = −0.10 in Experiment 1a participants], i.e., malingering participants with poorer spans showed a greater incidence of substitution errors. As a result, among participants with abnormal spans, an abnormal (*p* < 0.05) incidence of substitution errors showed 43% sensitivity and 91% specificity in distinguishing malingering subjects from controls.

**FIGURE 6:**
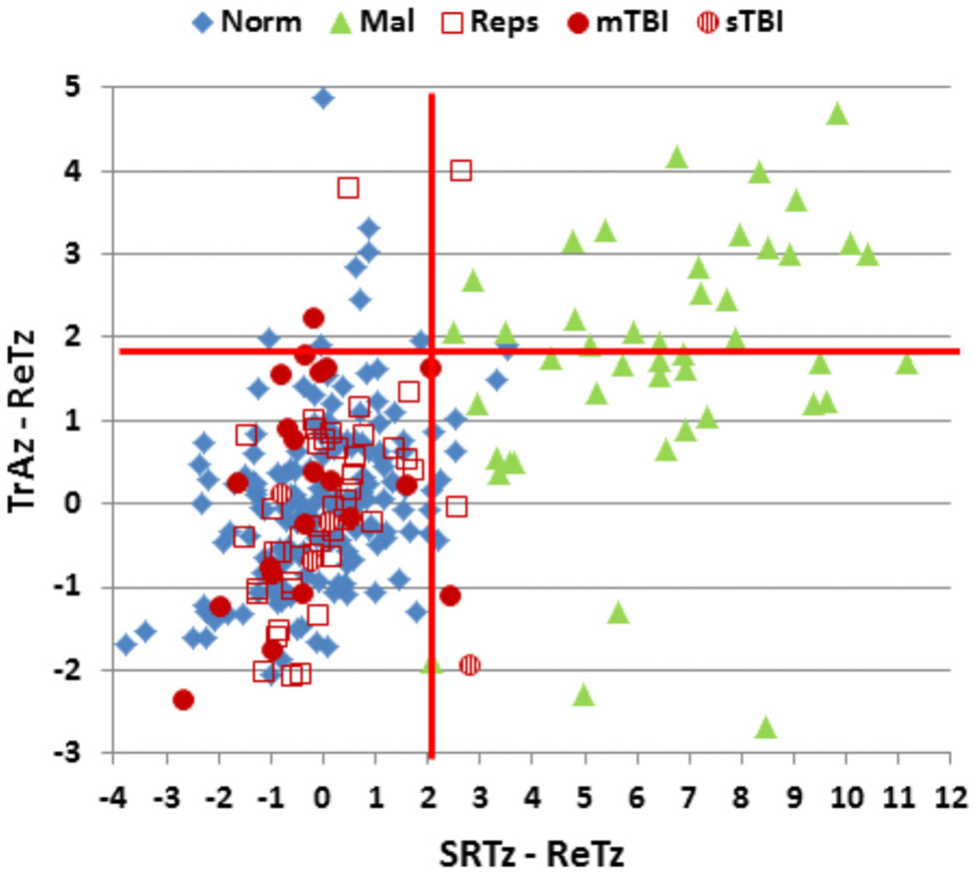
**Differences in ReT *z*-scores on the C-SST and two processing speed tests in malingering and control conditions.** Ordinate: Differences between ReT *z*-scores and *z*-scores on the Trail Making Test, part A (TrAz). Abscissa: Differences between Log ReT *z*-scores and Simple Reaction Time *z*-scores (SRTz). Horizontal and vertical red lines show the upper limits (*p* < 0.05) of the difference distribution in the normative control group of 187 participants (Norm). See **Figure 2** for group membership specifications.

Surprisingly, ReT *z*-scores in malingering subjects were similar to those of controls [ReT *z*-score = −0.09, *F*(1,234) = 0.28, NS], and did not differ from ReT *z*-scores in Experiment 1a [*F*(1,48) = 0.16, NS]. **Figure 6** shows the differences between ReT z-scores on the C-SST and *z*-scores on two other tests of processing speed, a simple reaction time test and the Trail Making Test, part A. Participants performing with full effort in the normative group (blue diamonds) and Experiment 1a (open red squares) showed similar processing speed *z*-scores across the three tests (the maximal group-wise *z*-score difference was 0.12 for the subjects in Experiment 1a). In contrast, the malingerers showed large delays on both the simple reaction time test (mean *z*-score = 6.43) and the Trail Making Test, part A (mean *z*-score = 1.63) while producing normal ReT *z*-scores on the C-SST.

We therefore examined the z-score differences between ReT *z*-scores, simple reaction time *z*-scores, and Trails A *z*-scores. All malingering subjects (100%) showed abnormal differences (*p* < 0.05, *z*-score difference >2.07) between ReTs and simple reaction time *z*-scores. In addition, 47% showed abnormal differences (*p* < 0.05, *z*-score difference >1.89) between ReT and Trails A *z*-scores in comparison to a 5% (by definition) false positive rate among controls. Considering only those subjects with abnormal MnS scores, 100% of malingering participants showed abnormal *z*-score differences with the simple reaction time test, and 68% showed abnormal differences with the Trail Making Test, part A (**Figure 6**). In contrast, none of the control subjects with abnormal spans showed abnormal differences between ReT *z*-scores and simple reaction time *z*-scores (i.e., sensitivity = 100%, specificity = 100%), while 18% showed abnormal differences between ReT *z*-scores and Trail Making Test part A *z*-scores (i.e., sensitivity = 68%, specificity = 82%).

### Discussion

Previous studies demonstrating reduced spatial span in malingering participants have suggested that performance cutoffs can distinguish between malingering and non-malingering populations (Bernard, 1990; Ord et al., 2008; Ylioja et al., 2009). We found that an MnS *z*-score cutoff (*p* < 0.05) provided 57% sensitivity and 95% specificity. However, when comparing the subgroups of malingering and control participants with abnormal spatial spans, we found that cutoff scores provided more modest sensitivity and specificity. For example, a *z*-score of 3.0 provided 36% sensitivity and 91% specificity among the participants with abnormal spans.

The incidence of substitution errors was increased in malingering subjects. However, a *p* < 0.05 cutoff based on the percentage of substitution errors showed a lower sensitivity (29%) than the MnS cutoff at a comparable level of specificity (95%). In contrast, sensitivity improved (to 43%) with little loss of specificity (93%) when only participants with abnormal MnS *z*-scores were considered. This suggests that an increase in the relative frequency of substitution errors can assist in confirming malingering in participants with abnormal spatial spans.

Finally, across-test comparisons of ReT *z*-scores and *z*-scores in other processing speed tests proved very sensitive to malingering. The C-SST was particularly useful in this regard, perhaps because malingering participants may have been unaware that processing speed was being incidentally measured along with item recall during C-SST testing. The difference between simple reaction time *z*-scores and ReT *z*-scores was within normal limits in 95% of control participants, and abnormal in 100% of malingering participants. In addition, ReT versus SRT *z*-score differences were within the normal range in all control subjects with abnormal spatial spans, and abnormal in all simulated malingerers with abnormal spans.

*Z*-score differences between ReTs and completion times on the Trail Making Test, part A also showed a sensitivity of 68% and a specificity of 82% in distinguishing between simulated malingerers and control participants with abnormal spans. Thus, *z*-score differences in ReTs and other processing-speed measures proved very sensitive in distinguishing between abnormal spans associated with simulated malingering and abnormal spans that occurred in participants performing with full effort.

### Limitations

The performance of simulated malingerers could be distinguished from that of controls with abnormal MnS scores based on performance cutoffs, error-pattern analysis, and comparisons of ReT *z*-scores with *z*-scores from processing speed tests. However, it remains unclear whether these comparisons would distinguish simulated malingerers from patients with clinical disorders (e.g., schizophrenia, mild cognitive impairment, etc.) who may show more behavioral variability and impairments of greater magnitude than control participants.

Unlike previous studies of the effects of simulated malingering on SSTs, the participants in Experiment 2 were familiar with the C-SST due to their participation in Experiment 1. Although repeated test exposure did not significantly influence performance in Experiment 1 (see above), the malingering strategies adopted by the participants in Experiment 2 may have been altered by their previous experience with the test. Further research with naïve malingerers is needed to address this issue.

## Experiment 3: The Effects of Traumatic Brain Injury

There is conflicting information surrounding the deficits in visuospatial memory that can occur following TBI. Wilde et al. (2004) found only insignificant differences in the spatial spans of 22 patients with TBI and a matched control group. However, deficits in recalling complex visual patterns have been reported following severe TBI (sTBI), particularly in the acute phase (Anderson and Catroppa, 2007), and deficits in recalling the spatial locations of visual patterns have been reported following mild TBI (mTBI) (Chuah et al., 2004). In general, however, TBI-related deficits in working memory appear to be more evident on verbal than visuospatial tasks (Anderson and Catroppa, 2007; Livengood et al., 2010), consistent with recent studies that find greater verbal than visuospatial memory deficits among veterans with mTBI and comorbid post-traumatic stress disorder (PTSD) (Vasterling et al., 2006; Sozda et al., 2014).

### Methods

#### Participants and Methods

The methods were identical to those used in the first session of Experiment 1. Thirty Veterans with a diagnosis of TBI following comprehensive neurological and neuropsychological examination were recruited from among such patients served by the Veterans Affairs Northern California Health Care System. Most of the patients had suffered one or more head injuries during recent military conflicts. All patients gave informed written consent following procedures approved by the Institutional Review Board of the VA Northern California Health Care System and were compensated for their participation. The patients included 29 males and one female between the ages of 20 and 61 years (mean age = 35.5 years), with an average of 13.6 years of education. All patients had suffered head injuries and a transient loss or alteration of consciousness, and had been clinically diagnosed after extensive neurological and neuropsychological examination. All were tested at least 1 year post-injury (range 1.3–15 years). Twenty-six of the patients had suffered one or more combat-related incidents, with a cumulative loss of consciousness of less than 30 min, no hospitalization, and no evidence of brain lesions on clinical MRI scans. These patients had been diagnosed with mTBI. The remaining four patients had suffered more severe accidents with hospitalization, coma duration exceeding 8 h, and post-traumatic amnesia exceeding 72 h. These patients had been diagnosed with sTBI. All patients were informed that the study was for research purposes only and that the results would not be included in their official medical records. Evidence of PTSD, as reflected in elevated scores (>50) on the Posttraumatic Stress Disorder Checklist (PCL), was evident in the majority of the TBI sample.

##### Exclusion of Malingering Patients

Two patients with mTBI, identified as performing with suboptimal effort in previous tests (Woods et al., 2015a,d,f), produced low MnS *z*-scores (-1.2 and −1.8, respectively). Both showed increased substitution errors and abnormal *z*-score differences between ReT *z*-scores and *z*-scores on the simple reaction time test. The data from both patients were therefore excluded from further analysis. **Table 1** provides overall demographic information for the 28 remaining patients with TBI included in Experiment 3, and **Table 3** provides additional information about TBI severity and etiology.

**Table 3:** Traumatic brain injury (TBI) patient characteristics.

ID	Age	Edu	Etiology	PT	PCL	MnS	MnDS	RT-z
PT001^c^	35	12	MVA	Severe	59	4.27	4.31	0.74
PT002^c,d^	24	12	Blast	Mild	54	6.20	6.50	−1.26
PT003^c,d^	28	12	Blast	Mild	66	4.67	6.23	0.34
PT005^d^	46	12	MVA	Severe	42	5.75	7.37	0.55
PT012^c,d^	57	16	MVA	Severe	56	4.30	4.79	1.02
PT014	30	14	MVA	Mild	–	4.90	6.67	−0.73
PT038^c^	52	18	MVA	Mild	27	4.00	6.83	−0.57
PT051^c,d^	41	14	Blast^a^	Mild	45	5.58	6.90	0.88
PT062	20	14	Blast^a^	Mild	41	6.67	7.83	−0.07
PT078^b,c^	46	14	MVA	Severe	46	5.57	7.08	1.26
PT081^d^	25	14	Fall	Mild	0	5.83	5.64	−0.62
PT101	28	13	Blast	Mild	47	6.17	6.83	−1.14
PT106^d^	25	14	Blast	Mild	57	4.25	5.42	0.25
PT109	29	10	Blast	Mild	54	6.17	7.08	0.44
PT110^c,d^	47	14	Blast^a^	Mild	52	4.40	6.30	0.35
PT111	28	12	Fall	Mild	43	5.40	8.70	0.09
PT112^c^	29	14	Blast	Mild	27	5.03	5.83	−0.94
PT113^d^	61	16	MVA^a^	Mild	52	5.83	4.79	0.13
PT114^c,d^	27	14	Blast	Mild	72	6.25	5.64	0.03
PT115^c,d^	48	13	Blast	Mild	59	4.33	5.57	0.75
PT117^c^	49	12	Fall	Mild	47	4.90	5.42	−0.01
PT120^c^	28	14	Fall	Mild	68	6.20	8.00	−0.66
PT122^c,d^	39	16	MVA	Mild	64	4.83	6.58	−0.16
PT123^c,d^	25	12	Blast^a^	Mild	72	3.82	4.10	0.28
PT125^c,d^	23	14	Fall	Mild	67	3.58	5.79	4.47
PT143^c,d^	29	14	Fall	Mild	47	4.42	8.08	1.40
PT174^c,d^	28	12	Blast	Mild	46	5.75	8.25	−0.82


*PT, traumatic brain injury severity; PCL, PTSD checklist; Age in years. Edu, years of education; MVA, moving vehicle accident.^a^Multiple TBIs. ^b^Female. ^c^Chronic Pain. ^d^Sleep Problems. Two patients who were rejected for suboptimal effort are not included.*

### Results

Performance summaries from the remaining patients are included in **Table 2** for mTBI and sTBI groups. MnS scores for individual mTBI (solid red circles) and sTBI (striped red circles) patients are included in **Figure 2**. MnS *z*-scores (**Figure 4**) were insignificantly reduced in the TBI group as a whole compared to the normative population [*z* = −0.25, *F*(1,213) = 1.51, NS], with similar insignificant reductions seen in the mTBI subgroup [*z* = −0.26, *F*(1,209) = 1.46, NS] and the small sTBI subgroup [*z* = −0.16, *F*(1,189) = 0.11, NS]. Although MnS *z*-scores in the TBI group were slightly reduced relative to the performance of the control subjects in Experiment 1a [*F*(1,75) = 4.93, *p* < 0.04, partial ω^2^ = 0.05], the effect size was small and further power analysis showed that 246 subjects would be required to provide a 95% chance of obtaining a difference with the Experiment 1a population at the *p* < 0.05 level. As shown in **Figure 5**, the types of errors made by patients with TBI resembled those committed by the control participants.

Mean patient ReTs are included in **Figure 3**. Patient ReT *z*-scores (**Figure 4**) showed minimal slowing in the TBI group as a whole [mean ReT *z*-score = 0.21, *F*(1,213) = 1.00, NS], though the ReT *z*-scores of the sTBI patient subgroup (mean *z*-score = 0.89) showed a weak trend toward increased ReT *z*-scores [*F*(1,189) = 3.16, *p* < 0.08]. However, power analysis showed that 1112 subjects would be required to have a 95% chance of obtaining a difference between sTBI patients and the normative population at the *p* < 0.05 level. *Z*-score differences in ReTs and processing-speed measures on other tests were also generally within the normal range (**Figure 6**).

### Discussion

After excluding two patients who showed evidence of malingering (Armistead-Jehle, 2010), we found no significant group-level abnormalities in the MnS scores of patients with TBI. These results are consistent with previous reports of preserved visuospatial span in TBI patient groups (Wilde et al., 2004) and contrast with group-level deficits in visuospatial span seen in other patient populations, including patients with schizophrenia (Chey et al., 2002), dementia (Wiechmann et al., 2011), and hemispatial neglect (Luukkainen-Markkula et al., 2011). We previously found significant abnormalities in digit span (*z*-score = −0.52) in the same TBI patient group (Woods et al., 2011b). One possible explanation is that TBI damage had greater effect on the anatomical regions subserving digit span (Baldo et al., 2012) than spatial span (Chechlacz et al., 2014). Alternatively, digit span may be more sensitive than spatial span to PTSD co-morbidity (Vasterling et al., 2006; Sozda et al., 2014), which was common in our patient group (see **Table 3**).

## Conclusion

We describe a computerized spatial span test (C-SST) that provides multiple measures of visuospatial memory span, including the maximal span metric of the Corsi Block Test, the total correct metric of the Wechsler Spatial Span Test, and the psychophysically based mean span (MnS) metric. The current experiments confirm that the MnS metric is a more precise measure of visuospatial memory than the other two metrics, with lower standard deviations and reduced coefficients of variation in comparison with traditional measures. In Experiment 1, the MnS showed higher test–retest reliability than the maximal span or total correct metrics. Experiment 1 also revealed that response times (ReTs) were highly reliable across tests, and that learning effects on both MnS and ReT scores were insignificant. Experiment 2 examined performance in simulated malingerers. MnS scores were abnormal in the majority of malingerers, but MnS *z*-score cutoffs were only partially successful in distinguishing simulated malingerers from control participants, particularly among those with abnormal spans. The percentage of substitution errors also increased in malingering, enabling simulated malingerers to be discriminated from control participants with abnormal spans with moderate sensitivity and specificity. In addition, because ReTs did not increase in simulated malingerers, in contrast to the pronounced slowing seen on other tests of processing speed, *z*-score differences in processing speed measures enabled the discrimination of simulated malingerers and controls, even those with abnormal spans, with high specificity and sensitivity. Experiment 3 examined performance in patients with TBI. Consistent with previous reports, no significant group-level abnormalities were found. The C-SST enhances the reliability and sensitivity of spatial span testing, assists in detecting poor performance due to malingering, and provides precise measures of visuospatial memory in patient populations.

## Conflict of Interest Statement

The authors declare that the research was conducted in the absence of any commercial or financial relationships that could be construed as a potential conflict of interest. David L. Woods is affiliated with NeuroBehavioral Systems, Inc., the developers of Presentation software that was used to create these experiments.

## Footnotes

1 The CCAB included the following computerized tests and questionnaires: finger tapping, simple reaction time, Stroop, digit span forward and backward, verbal fluency, verbal list learning, spatial span, trail making, vocabulary, design fluency, the Wechsler Test of Adult Reading (WTAR), visual feature conjunction, risk and loss avoidance, delay discounting, the Paced Auditory Serial Addition Task (PASAT), the Cognitive Failures Questionnaire (CFQ), the Posttraumatic Stress Disorder Checklist (PCL), and a traumatic brain injury (TBI) history and symptom questionnaire.

2 An open source executable version of the C-SST can be downloaded at http://www.ebire.org/hcnlab/.
